# Cognitive and Functional Deterioration in Patients with Severe COPD^1^

**DOI:** 10.1155/2006/848607

**Published:** 2006-07-17

**Authors:** Cengiz Özge, Aynur Özge, Özgür Ünal

**Affiliations:** ^1^Department of Chest DiseaseMersin University School of MedicineMersinTurkey; ^2^Department of NeurologyMersin University School of MedicineMersinTurkey

**Keywords:** Chronic obstructive pulmonary disease (COPD), cognitive impairment, functional impairment, hypoxemia, hypercarbia

## Abstract

The objective of this study was to examine the association among the duration of COPD, degree of hypoxemia, and neurological abnormalities including cognitive functioning. Fifty-four patients with severe COPD and 24 age- and sex-matched controls, were included in the study. All patients and controls were administered pulmonary function tests, standardized Mini-mental State Examination (MMSE), Blessed Dementia Scale (BDS), Physical Self-maintenance Scale (PSMS), Modified Activities of Daily Living scale (MADL), Instrumental Activities of Daily Living scale (IADL), Cornell Scale for Depression in Dementia (CSDD), Global Deterioration Scale (GDS) and Clinical Dementia Rating (CDR). In addition, detailed physical and neurological examinations were performed. Sixty-four percent of patients with COPD showed abnormalities in MMSE, predominantly in recent memory, construction, attention, language, and orientation domains. Functional abnormalities were correlated with cognitive abnormalities. Although COPD patients did not show significant depression compared to controls, 77.7%. of the patients showed subjective and objective cognitive disturbance and 72.2% of the patients were classified as questionable or mild dementia. In conclusion, patients with COPD show significant cognitive and functional impairments that cannot be explained just by coincidence or by depression.

## 1. Introduction

Chronic obstructive pulmonary disease (COPD) is a disease state characterized by airflow limitation that is not fully reversible. The airflow limitation is usually both progressive and associated with an abnormal inflammatory response of the lungs to noxious particles or gases, which leads to hypoxemia and hypercarbia due to poor ratio of ventilation to perfusion in the lung parenchyma [11].

Our first objective was to identify the neurological disturbances, especially cognitive and functional impairment, in patients with COPD. We hypothesized that patients with documented severe hypoxia would have significantly more neurological problems than those classified as mildly hypoxic. Although the age-related decline in cognitive functioning of healthy adults is a well-documented phenomenon, there is a strong relationship between COPD and cognitive dysfunction [19, 20,25]. Additionally, patients with COPD generally showed a high frequency of cognitive impairment possibly related to duration and severity of hypoxemia and affective status of patients [7,24,25]. Some studies showed that 27% of patients with mild hypoxemia and 61% of patients with severe hypoxemia demonstrated neuropsychological deficits [17]. Our second objective was to investigate the association of hypoxia and the parameters of expiratory airflow limitation with cognitive and daily functioning.

ISSN 0953-4180/06/$17.00 © 2006 – IOS Press and the authors. All rights reserved

## 2. Study design and patients

### 2.1. Subjects

This was a prospective clinical and neuropsychological study performed with collaboration of the Departments of Chest Disease and Neurology at the Hospital of Mersin University School of Medicine. The study population included 54 patients with severe COPD, and 24 age- and sex-matched subjects as the control group. Written informed consent was obtained from all subjects. All participants were right-handed as indicated by positive laterality quotients on the Edinburgh Handedness Inventory [27]. Both the study and the control subjects underwent ophthalmologic and audiologic examinations to exclude visual or hearing deficits, which might compromise the reliability and validity of the neuropsychological testing. Chest X-ray, pulmonary function tests (PFT), arterialized blood sample analysis, neuropsychological screening, electroencephalography (EEG) and cranial computed tomography (CT) were performed in all subjects.

Inclusion criteria for the study were as follows: 1) strict diagnosis of COPD according to GOLD criteria [11], 2) no presence of predicted memory or language deficit, 3) being free of any psychotropic drugs for at least 2 weeks prior to the study, 4) being neurologically intact (no epilepsy, stroke, head injury, dementia, toxic chemical exposure, sleep disorder, etc), 5) no history of concomitant severe or chronic medical illnesses (hepatic failure, chronic renal failure, decompensate diabetes mellitus etc.) 6) no history of depression and other major psychiatric disorders (anxiety disorders, schizophrenia, substance or alcohol abuse/dependence) according to DSM-IV criteria [1], 7) no history of psychosurgery or any other neurosurgical procedure.

Standardized pulmonary function tests were performed using dry spirometer device (Sensor Medics MPM, Yorba Linda, CA). We calculated the forced expiratory volume in 1 second ($FEV_1$), the forced vital capacity (FVC), and $FEV_1$/FVC ratio from maximal expiratory maneuvers. Data were expressed as a percentage of the predicted standardized values according to European Respiratory Society (ERS) criteria [23]. The acceptable recordings were obtained from each maneuver and the highest values were used for further analysis [2,28].

Arterial blood gas analysis was performed by using ABL 330. $PaO_2$, $O_2$ saturation, pH and $HCO_3$ were evaluated for each patient. Patients with $FEV_1$ levels lower than 50% were classified as having severe or very severe COPD according to GOLD criteria [11].

All patients received appropriate treatment for COPD. At the time of the study, patients were receiving a standardized daily therapy including the following medications: long acting $\beta$2-agonist (24 patients- 44.4%), long acting $\beta$2-agonist and long-acting anti-cholinergic drugs (19 patients- 35.1%), long acting $\beta$2-agonist combined with long-acting anti-cholinergic drugs and theophylline (11 patients- 20.5%). None were receiving inhaled or systemic corticosteroid therapy. Blood levels of theophylline were in the therapeutic ranges in all patients receiving the drug. All patients had stable COPD according to GOLD criteria [11], and none of them had carbon dioxide narcosis or encephalopathy according to clinical or EEG data. A detailed history of COPD and memory deficit were inquired and recorded with a structured interview. Physical and neurological examinations were completed for each subject by the same neurologist (ÖÜ).

### 2.2. Neuropsychological assessment

Standardized MMSE Test [9] was used to evaluate cognitive status. This instrument explores spatial and temporal orientation, short- and long-term verbal memory, attention, verbal attainment, and practical abilities in 12 items and 30 questions. The correct answer to each question is scored with 1 point (total from 0 to 30). A score less than 24 indicated a generic cognitive dysfunction.

The Blessed Dementia Scale (BDS) [12] was designed to assess quantitatively the signs of dementia to enable comparisons with pathological changes. The scale is divided into two: the information-memory-concentration test and the dementia scale, which incorporated changes in everyday performance habits and personality, and assessed orientation, memory and concentration, which have been shown to differentiate groups of demented and non-demented aged persons. The total score lies between 0 (fully preserved capacity) and 28 (extreme incapacity).

Cornell Depression Scale in Dementia (CSDD) [14] is a scale for the diagnosis of depression in patients with a dementia syndrome. The 19-item scale is rated on a three-point score of absent, mild or intermittent and severe with a note when the score was unavailable. A score of 8 or more suggests significant depressive symptoms.

Activities of Daily Living Scale is a battery to assess the functional ability of older people in relation to ac-

Table 1
Demographic characteristics of the patients with COPD and healthy volunteers

| | Patients with COPD ($n = 54$) | Controls ($n = 24$) |
|---|---|---|
| Age (years) (mean ± SD) | 64.6 ± 8.5 | 62.4 ± 8.4 |
| Gender (Female) n (%) | 8 (14.8%) | 7 (29.2%) |
| Years of formal education (mean ± SD) | 5.1 ± 4.8 | 6.7 ± 4.5 |
| Actual smokers (%) | 31.4 | 8.3 |
| Patients with hypertension (%) | 11.1 | 8.3 |
| Patients with diabetes mellitus (%) | 1.9 | None |
| Patients with peripheral arterial disease (%) | 1.9 | None |
| Family history of | | |
| COPD (%) | 24.1 | 8.3 |
| Malignancy (%) 9.3 | 8.3 | |
| Stroke (%) | – | 12.5 |
| Dementia (%) | 7.4 | 4.1 |
| Coronary artery disease (%) | 11.1 | 20.8 |

tivities of daily living [21]. Three scales are as follows; Physical Self-maintenance Scale (PSMS), Instrumental Activity of Daily Living scale (IADL), and Modified Activities of Daily Living scale (MADL). PSMS details basic self-care tasks and its score lies between 6 (fully preserved capacity) and 30 (extreme incapacity). IADL concerns more complex daily tasks and its score lies between 8 (fully preserved capacity) and 31 (extreme incapacity). MADL comprises standardized daily living activities and its score lies between 0 (full preserved capacity) and 16 (extreme incapacity).

Global Deterioration Scale (GDS) [3] is a staging instrument indicating deterioration in dementia. The GDS is made up of detail clinical description of seven major clinically distinguishable stages, ranging from normal cognition (score 1) to very severe dementia (score 7). Current validity of the GDS was demonstrated by a highly significant correlation with other neuropsychological tests such as the MMSE.

Clinical Dementia Rating (CDR) [5] is usually completed in the setting of a detailed knowledge of the individual patient. CDR has now become one of the gold standards of global rating of dementia in trials of patients with Alzheimer's disease. CDR ratings are 0 for healthy people, 0.5 for questionable dementia, and 1, 2 or 3 for mild, moderate and severe dementia as defined in the scale.

### 2.3. Electroencephalographic tests

Each subject was seated in a soundproof, light-controlled, well ventilated recording room, while 30 minutes of resting EEG data were being collected from the 14 monopolar electrode sites of the International 10/20 system, referred to as Cz. Computerized EEG recordings were obtained using Medelec Profile 40-channel digital EEG equipment (Oxford, England). The electrode impedance was carefully kept below 5 k$\Omega$. Hyperventilation was employed as standardized activation method. The filtering interval, frequency and amplitude of the device were adjusted to 10 to 59 Hz, 30 mm/sec, and 100 $\mu$V, respectively. The EEG results were evaluated visually.

### 2.4. Statistical analysis

Data were first evaluated using descriptive statistics. Differences in clinical and neuropsychological parameters between patients and control groups were assessed by parametric and non-parametric statistics, as appropriate. Pearson and Spearman Correlation analyses were run among clinical, spirometric and neuropsychological variables in patient group, as appropriate. In order to investigate the effect of clinical and spirometric variables on the cognitive and functional performance, linear regression analysis by Enter method was used. Values of $p < 0.05$ were considered statistically significant.

## 3. Results

### 3.1. Subjects

Demographic features of the study groups are shown in the Table 1. COPD and control subjects were statistically similar with respect to gender, age and the duration of formal education. Table 1 also shows the mean duration of COPD and some related health information both in the personal and family history of the study subjects.

Table 2
Pulmonary testing results of patients with COPD

| Pulmonary function test parameters | Mean ± SD |
|---|---|
| Forced Expiratory Volume (FEV1) (L) | 0.98 ± 0.35 |
| Forced Expiratory Volume (FEV1) (%) | 35.3 ± 11.5 |
| Forced Vital Capacity (FVC) (L) | 1.98 ± 0.88 |
| Forced Vital Capacity (FVC) (%) | 45.7 ± 10.8 |
| FEV1/ FVC ratio | 52.8 ± 16.4 |
| Partial pressure $O_2$ (mmHg) | 47.3 ± 2.6 |
| Partial pressure $CO_2$ (mmHg) | 44.3 ± 6.5 |
| pH | 7.36 ± 0.06 |

### *3.2. Pulmonary testing*

Values shown in the Table 2 correspond to severe or very severe airflow limitation according to GOLD criteria. The mean $PaO_2$ of 47.3 $\pm$ 2.6 mmHg was below the normal range of 80 to 100 mmHg, indicating the presence of moderate hypoxia in our sample. Consistent with this finding, the mean $PaCO_2$ of 44.3 $\pm$ 6.5 mmHg was slightly above the normal range of 35 to 45 mmHg, which indicates hypercapnea of moderate degree.

### *3.3. Neurological examination*

Neurological examination revealed the following: bilateral resting tremor in 4 patients (7.4%), positive palmomental reflex in 7 patients (12.9%), and decreased tendon reflexes in 2 patients (3.7%).

Five patients in the COPD group and one in the control group showed a specific fronto-temporal slow wave pattern on EEG, especially in the left cerebral hemisphere; however, none had epileptiform EEG abnormalities and significant amplitude asymmetry. One patient showed low amplitude changes, especially in the frontal areas (see Fig. 1). None of the subjects revealed encephalopathic or sleep changes during resting EEG.

Cranial CT scans without contrast were obtained for 31 of 54 participants (57.4%). None of the patients showed important CT abnormality, except for non-specific ischemic changes especially in the periventricular regions. None of the neuroimaging examination suggested degenerative (e.g. Alzheimer disease) or multi-infarct dementia.

## 4. Neuropsychological findings

Thirty-five patients with COPD (64.8%) showed MMSE abnormalities. The mean score of MMSE was 24.6 $\pm$ 3.7 (13 to 30) in COPD patients, which was significantly lower compared to that of healthy controls (27.3 $\pm$ 1.9) ($p = 0.0001$). None of the control subjects showed clinically important MMSE abnormalities. Observed MMSE abnormalities were predominantly in recent memory (25.9%), construction (38.8%), attention (31.4%), language (12.9%), and orientation (24.0%) (some examples of drawing abnormalities are shown in Fig. 2). Five of the patients (9.2%) who reported subjective complaints of memory and whose MMSE scores were less than 24, showed generalized disturbance in all domains. The mean score of the BDS was 9.6 $\pm$ 6.6 (from 0 to 25), and 27 patients (50%) showed increased BDS score (higher than 6 points, represented by 2 standard deviation over the mean control values). For the two patients who reported depression and questionable dementia, the results of the BDS were generally consistent with the MMSE results (see Table 3).

Patients with COPD were not significantly more depressed than controls, as measured with CSDD scores (6.2 $\pm$ 5.1 vs 4.5 $\pm$ 4.2, $p = 0.130$). Fourteen (25.9% of) patients with severe COPD showed a depression score above the cut-off value of 8 points. Five patients out of 14 who had depression as measured with the CSDD showed no significant cognitive impairment on the MMSE and BDS. Depression was not associated with cognitive impairment in these five patients.

When 2 SD over the mean scores of the controls were accepted as abnormal (95% confidence interval), rates of abnormal scores in functional capacity scales were as follows: 14.8% in MADL (8 patients), 22.2% in IADL (12 patients) and 20.3% in PSMS (11 patients). Except for one patient who only showed a higher depression score, all patients with abnormal scores on functional capacity, also had significant degree of cognitive impairment as detected by both the MMSE and the BDS.

Staging of the dementia showed normal findings in 12 patients (22.2%) by the GDS and 15 patients (27.7%) by the CDR. Other patients showed questionable or mild dementia by those scales (see Table 4). One patient who had increased GDS score and 7 patients (12.9%) who had questionable dementia by CDR, did not show important cognitive impairment on both the MMSE and the BDS. Four of these patients showed increased CSDD score but others showed no important cognitive or functional impairment by the test battery used.

Correlation analyses among clinical, spirometric and neuropsychological data yielded some relationships. Age was negatively correlated with CSDD score (CC:0.33, $p = 0.013$) and hypoxemia correlated with the frequency of sleep disturbances. On the other hand,

Table 3
Results of neuropsychological and functional screening

| | Patients with COPD $N = 54$ | Controls $n = 24$ | $p$ |
|---|---|---|---|
| MMSE | $24.6 \pm 3.7$ | $27.3 \pm 1.9$ | 0.0001 |
| BDS | $9.6 \pm 6.6$ | $4.9 \pm 4.5$ | 0.001 |
| MDLA | $0.3 \pm 0.8$ | $0.04 \pm 0.2$ | 0.036 |
| IDLA | $0.72 \pm 1.7$ | $0.1 \pm 0.6$ | 0.030 |
| PSMS | $6.4 \pm 1.1$ | $6.0 \pm 0.0$ | 0.005 |
| CSDD | $6.2 \pm 5.1$ | $4.5 \pm 4.2$ | 0.130 |
| GDS (n, %) | | | 0.511 |
| Stage 1 | 12 (22.2%) | 9 (37.5%) | |
| Stage 2 | 28 (51.8%) | 11 (45.8%) | |
| Stage 3 | 9 (16.6%) | 3 (12.5%) | |
| Stage 4 | 5 (9.4%) | 1 (4.1%) | |
| CDR (n, %) | | | 0.000 |
| Normal | 15 (27.7%) | 18 (75.0%) | |
| Questionable dementia | 34 (62.9%) | 6 (25.0%) | |
| Mild dementia | 5 (9.2%) | 0 | |

MMSE: Mini-mental state examination test, BDS: Blessed dementia scale, MADL: Modified activities of daily living scale, IADL: Instrumental activities of daily living scale, PSMS: Physical self- maintenance scale, CSDD: Cornell scale for depression in dementia, GDS: Global deterioration scale and CDR: Clinical dementia rating.

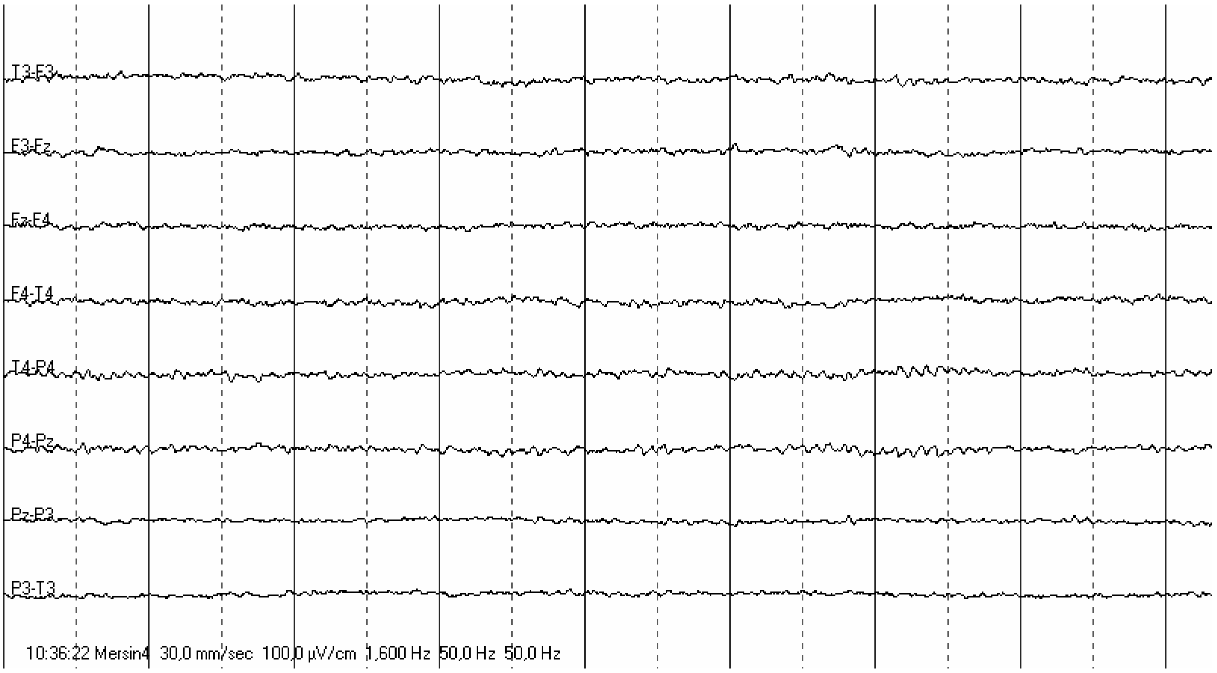

Fig. 1. The low amplitude pattern of EEG during resting state in a patient with severe COPD.

hypercarbia was correlated with cognitive impairment (BDS score), functional impairment (IADL score), depressive mood (CSDD), clinical stage of cognitive disturbance (CDR) and the frequency of sleep disturbances (see Table 4). Among spirometric variables, $FEV_1$ (%) was correlated with MMSE, MADL and PSMS scores. FVC (%) was correlated with general cognitive impairment (MMSE). $FEV_1$/FVC was correlated with the cognitive scores on both the MMSE and the BDS. Although none of these correlations were significant after applying the Bonferroni correction for multiple comparisons, there were significantly more correlaa) b) c)

Fig. 2. Some examples of the drawings of one healthy control (a) and patients with COPD (b and c).

tions with an alpha level of $p < 0.05$ than would be expected by chance (chi square $= 5.7$; $p = 0.017$). There were significant correlations between MMSE and other tests used in the study, even after applying the Bonferroni correction for multiple comparisons [i.e. BDS (CC:0.80, $p < 0.001$), MADL (CC:0.46, $p < 0.001$),

Table 4
Correlations between neurological dysfunctions and pulmonary function tests

| | $PaO_2$ | $PaCO_2$ | pH | FEV1 (%) | FVC (%) | FEV1/FVC |
|---|---|---|---|---|---|---|
| MMSE | – | – | CC:0.33 $p = 0.01$ | CC:0.33 $p = 0.005$ | CC:-0.14 $P = 0.02$ | CC:-0.11 $p = 0.03$ |
| BDS | – | CC:0.28 $p = 0.03$ | CC:-0.27 $p = 0.04$ | – | – | CC:0.19 $p = 0.05$ |
| MADL | – | – | – | CC:-0.72 $p = 0.01$ | – | – |
| IADL | – | CC:0.30 $p = 0.02$ | – | – | – | – |
| PSMS | – | – | – | CC:-0.11 $p = 0.01$ | – | – |
| CSDD | – | CC:0.33 $p = 0.02$ | CC:-.28 $p = 0.04$ | – | – | – |
| GDS | – | – | CC:-.28 $p = 0.04$ | – | – | – |
| CDR | – | CC:0.30 $p = 0.02$ | – | – | – | – |
| Sleep disorder | CC:0.50 $p = 0.008$ | CC:0.47 $p = 0.006$ | – | – | – | – |

MMSE: Mini-mental state examination test, BDS: Blessed dementia scale, MADL: Modified activities of daily living scale, IADL: Instrumental activities of daily living scale, PSMS: Physical self- maintenance scale, CSDD: Cornell scale for depression in dementia, GDS: Global deterioration scale and CDR: Clinical dementia rating. CC: Correlation coefficient.

IADL (CC:0.47, $p < 0.001$), PSMS (CC:0.46, $p < 0.001$), GDS (CC:0.62, $p < 0.001$) and CDR (CC:0.72, $p < 0.001$)].

When the cognitive and functional scores were entered as independent variables, and spirometric and basic clinical parameters as dependent variables, the results of the linear regression analysis showed a significant effect of formal education years on the MMSE ($p = 0.001$), BDS ($p = 0.001$), MDL ($p = 0.039$), IADL ($p = 0.028$), CSDD ($p = 0.013$) and CDR ($p = 0.001$) scores. Only the $FEV_1$/FVC ratio showed a significant effect on the CSDD score ($p = 0.030$) in this model.

## 5. Discussion

This prospective clinical and neuropsychological study showed an increased frequency of cognitive and functional impairments in patients with severe COPD. These results indicate a possible effect of chronic hypoxemia on cognitive function and functional capacity. Cognitive and functional impairments were correlated with hypercarbia and expiratory airflow limitation. Our results might explain the decreased quality of life and increased cognitive disturbances in these subjects. To our knowledge, this is the first study about the effects of chronic hypoxemia on the cognitive and functional performance of patients with moderate to severe COPD. Results of this study suggest that the patients with COPD show significant cognitive and functional impairment.

Our results are consistent with previous studies [13, 15]. Stuss et al. [7] and Grant et al. [17] found a strong relationship among current neuropsychological measures, decreased oxygen partial pressure ($PaO_2$), and increased carbon dioxide partial pressure ($PaCO_2$). Memory dysfunction related to hypoxia is perhaps due to damage to the limbic memory region [7]. Another important study showed diffuse mental deterioration in COPD population, with particular impairment of higher cortical functions. Verbal performance and semantic memory were affected more than other cognitive domains [16]. We showed an increased frequency of important disturbances in recent memory, construction, attention, language, and orientation, complementing previous reports [7,16,17].

There are three possible anatomic/pathologic bases for the attention disorder noted in this sample. First, prolonged oxygen deprivation might result in a chronic state that would resemble the effects of acute deprivation as demonstrated in laboratory research. Second, the hypercapnea may produce a chronic sleepiness, affecting attention performance. And third, prolonged oxygen deprivation may lead to more permanent diffuse brain damage, affecting general attention capacity. Alternatively, there may be more direct involvement of "frontal system", perhaps implicating anterior attentional systems [6]. A suggestive comprehensive study by Incalzi et al. [26] compared the neuropsychological and cerebral perfusion performance of moderately or severely hypoxemic COPD patients to patients with Alzheimer Disease (AD). They reported that, opposite to the expected, cognitive scores of patients with COPD were lower than patients with AD. Authors concluded that anterior cerebral hypoperfusion and selected neuropsychological dysfunctions characterized hypoxemic COPD patients could herald frontal-type cognitive decline with the worsening of the hypoxemia.

Stuss et al. [6] hypothesized that significant impairments in anterograde memory deficit might be due to the sensitivity of the hippocampal or general limbic memory region to hypoxia [30]. Also, it was reported that both defective retrieval and inaccurate recognition affect verbal memory of COPD patients [25]. As opposed to these results, we did not find a significant relationship between cognitive disturbance and severe hypoxemia but a significant relationship between cognitive impairment and decreased FEV% levels. These contrasting results might be explained by differences in inclusion criteria and demographic features of the patients. We think that $FEV_1$% levels are more informative than $PaO_2$ levels for the purpose of evaluating chronic central nervous system effects of hypoxemia in patients with severe COPD.

Some authors observed that physiological, functional, and psychological consequences of COPD are only weakly related to each other [4,22]. Older individuals who have COPD may be affected in three ways that are relevant to cognitive functioning. They may experience age-related declines in blood flow, disease-related decline in arterial oxygen content, and both age- and disease-related decline in physical activity [18]. The combination of three factors may affect cognitive and functional capacity, especially at advanced stages of the disease. Perfusion studies show that chronic hypoxemia and expiratory airflow limitations are associated with widespread reduction in cerebral blood flow. Frontal and subcortical areas are most affected. Like patients with Alzheimer's disease, patients with advanced COPD were found to experience a high prevalence of forgetfulness due to retrieval deficits and impaired executive functions, probably because of the worsening of frontal and/or subcortical metabolism [26]. Although our study did not include a perfusional test, the results of neurological (tremor, palmo-mental reflexes, etc.) and neuropsychological examination (orientation, attention, construction deficit, etc.) highly suggested a possible dysfunction of frontal and subcortical circuits. EEG and CT examinations of some patients also supported this hypothesis. In addition to cognitive dysfunction, detected functional impairment in daily living activities in our patients were possibly related with these anatomical correlations.

Activity of left frontal area is highly involved in the regulation of attention and verbal production, and is correlated with the average cognitive performance, as reflected by the number of correctly performed cognitive tasks. Some reports pointed to a high prevalence of frontal type behavioral and cognitive problems in advanced COPD [16]. In this study, we investigated only severe or very severe COPD patients and determined an important degree of cognitive impairment especially in the areas of frontal dysfunction, particularly of the left side. Left frontal dysfunction might also account for the high prevalence of depression observed in this group of patients.

As previously demonstrated by Incalzi et al. [25], a close parallelism exists between cognitive decline and development of depressive symptoms in patients with COPD. The combination of cognitive and affective symptoms is quite common in geriatric populations. Patients with organic dementia frequently also become depressed, and primarily depressed patients show cognitive changes severe enough to be referred as pseudodementia. Although it was not the primary aim of this study, we diagnosed 5 patients (9.2%) with pseudodementia and others (16.6%) with a disturbance of affective status. Discriminant analysis did not show any specific cognitive impairment related to depressive symptoms in patients with severe COPD. Considering the patients who had cognitive impairment without depression (26 patients, 48.1%), we think that our results of cognitive and functional impairment could not be explained just by depression of chronic hypoxemia and it might be related with other mechanisms explained above.

Which factor is the most important in the occurrence of cognitive or functional disturbances in patients with severe COPD? Some authors suggested that the duration of COPD rather than the actual severity of chronic respiratory failure accounts for cognitive deterioration [24]. In this study, correlation and regression analysis results showed an important relationship between the cognitive impairments and the airflow limitation parameters. Some previous reports partly suggested these findings [10,18] and also pointed out a special relationship between the pulmonary function tests (i.e: $FEV_1$ %) values and working memory span. The sensitivity of these functional and cognitive domains to hypoxia was predicted based on pathologic considerations. Previously published results have varied from virtually no correlation between various measures of hypoxia and neuropsychological functioning [29] to low or modest but reliable correlations [8,13]. With the exception of Hupper's study [10], who found correlations of 0.75 to 0.82 with memory, the statistical relationships reported between cognitive functioning and hypoxia has been surprisingly low. Our study suggested the importance of poor airway parameters and co-morbid affective problems both on the cognitive and functional impairments in patients with moderate or severe COPD.

The strenghts of this study are: (1) prospective clinical and neuropsychological study design, (2) detailed neurological examination, EEG and neuroimaging of the patients with cognitive or other neurological complaints, (3) evaluation of both cognitive and functional capacity.

Basic limitations of the present study are as follows: (1) study design that did not evaluate longitudinal changes in the disease and cognitive disturbances, (2) lack of perfusion study of the brain, (3) lack of polysomnographic records of the patients in order to determine associated sleep disturbances. However, these limitations do not weaken the main message from the present study: Cognitive and functional decline frequently occurs in patients with severe COPD and are highly prevalent in subjects, especially who have low education, severe bronchial obstruction, moderate hypercarbia rather than hypoxemia, previous problems in daily living activities, and coexisting depressive symptoms. These data are important in the planning of future management and social strategies for these patients.
